# 手术前后血清CEA和CYFRA21-1水平的变化有助于预测非小细胞肺癌患者的预后

**DOI:** 10.3779/j.issn.1009-3419.2015.06.05

**Published:** 2015-06-20

**Authors:** 新春 段, 永 崔, 民 龚, 峰 田, 管 侍, 炳群 吴, 明亮 柳, 佳运 郭, 媛媛 孔

**Affiliations:** 100050 北京，首都医科大学附属北京友谊医院胸外科 Department of Toracic Surgery, Beijing Friendship Hospital, Capital Medical University, Beijing 100050, China

**Keywords:** 肺肿瘤, CEA, CYFRA21-1, 手术, 总生存时间, 预后, Lung neoplasms, CEA, CYFRA21-1, Surgery, OS, Prognosis

## Abstract

**背景与目的:**

血清癌胚抗原（carcinoembryonic antigen, CEA）和细胞角蛋白19的可溶性片段（soluble fragment of cytokeratin-19, CYFRA21-1）系非小细胞肺癌（non-small cell lung cancer, NSCLC）患者术前检查重要的肿瘤标志物（tumor markers, TMs），但其对NSCLC患者术后的预后作用尚存争议。本研究旨在探讨血清CEA和CYFRA21-1在手术治疗后的NSCLC患者预后中的临床价值。

**方法:**

回顾性总结175例经手术并辅以化疗的NSCLC患者的临床资料及随访情况，依据CEA、CYFRA21-1水平进行分组，用*Kaplan-Meier*法对各组进行生存分析。用*Cox*比例风险回归模型分析影响NSCLC患者术后预后的因素。

**结果:**

术前CEA、CYFRA21-1升高组的患者总生存时间（overall survival, OS）少于术前正常组的患者，术前CYFRA21-1升高组差异有统计学意义（*P*=0.001）。与术前术后CEA、CYFRA21-1均正常等组的患者OS比较，术前术后CEA、CYFRA21-1均升高组的患者OS最短，两组差异均有统计学意义（*P* < 0.05）。与CEA联合CYFRA21-1术前术后均正常等组的患者OS比较，CEA联合CYFRA21-1术前术后均升高组的患者OS最短，差异有统计学意义（*P* < 0.001）。CEACYFRA21-1（HHHH）、CEACYFRA21-1（NNHH）、CYFRA21-1（HH）、CEA（HH）、男性是判断预后的独立危险因素（*P* < 0.05）。

**结论:**

血清CEA或CYFRA21-1在手术前及术后均高于正常，尤其是两者联合在手术前及术后均升高的NSCLC患者预后不良。手术前后血清CEA、CYFRA21-1的检测有助于NSCLC患者术后预后的判断。

## Abstract (en)

**Background and objective:**

Serum carcinoembryonic antigen (CEA) and the soluble fragment of cytokeratin 19 (CYFRA21-1) are important tumor markers (TMs) in the preoperative examination of patients with non-small cell lung cancer (NSCLC). However, the prognostic role of these markers in NSCLC patients remains controversial. The aim of the study was to investigate the clinical significance of serum CEA variances and CYFRA21-1 levels for the prognosis of NSCLC patients following surgery.

**Methods:**

This retrospective study investigated the clinical records and follow-up sessions of 175 patients with NSCLC who accepted surgery and adjuvant chemotherapy. Patients were subdivided into groups based on serum CEA and CYFRA21-1 levels. Survival analysis was conducted using *Kaplan-Meier* method for each group. The prognostic factor was evaluated using *Cox* proportional hazards model.

**Results:**

The overall survival (OS) of patients with high preoperative CEA or CYFRA21-1 levels was lower than that of patients with normal preoperative CEA or CYFRA21-1 levels. The OS displayed a significant difference (*P*=0.001) between groups with high and normal preoperative CYFRA21-1. Compared with groups exhibiting normal preoperative and postoperative levels of CEA or CYFRA21-1, the OS was shorter for groups with high preoperative and postoperative levels of CEA or CYFRA21-1. The difference of the paired groups was significant (*P* < 0.05). Compared with the groups with normal preoperative and postoperative levels of CEA and CYFRA21-1, the OS was lower for the groups with high preoperative and postoperative levels of CEA and CYFRA21-1, which indicated a significant difference (*P* < 0.001). The CEACYFRA211 (HHHH), CEACYFRA211 (NNHH), CYFRA21-1 (HH), CEA (HH), and male gender were identified as independent prognostic factors (*P* < 0.05).

**Conclusion:**

This study suggested that the prognosis of NSCLC patients was not significantly satisfactory if preoperative and postoperative level of serum CEA or CYFRA21-1 was higher than standard value, especially if the preoperative and postoperative levels of CYFRA21-1 and CEA were higher than the standard values. The measurement of preoperative and postoperative levels of CYFRA21-1 and CEA proved helpful for the prognosis of patients with NSCLC.

目前为止，手术依然是治疗肺癌最主要的、最标准的治疗方式^[1-4]^，不容忽视的是，肺癌术后患者的OS差别明显，据报道^[2, 4]^Ia期患者5年生存率约71%-77%，Ib期患者约60%。因此，了解肺癌术后患者预后情况，对预后差的患者重点监测，同时给予积极的抗肿瘤综合性治疗，有益于提高患者术后生存率。

大量研究^[1-3, 5-7, 14-22]^表明，肿瘤标志物（tumor markers, TMs）可以预测非小细胞肺癌（non-small cell lung cancer, NSCLC）患者的预后，普遍的结论是TMs水平升高的患者比正常的患者预后差。多项研究的共同点是突出了术前术后均升高组的患者预后最差，并证实癌胚抗原（carcinoembryonic antigen, CEA）（HH）是判断预后的独立危险因素^[1-3, 7]^，但他们仅对CEA进行了研究。多项研究^[5, 6]^揭示细胞角蛋白19的可溶性片段（soluble fragment of cytokeratins-19, CYFRA21-1）同样对NSCLC患者术后预后具有预测作用。本研究采用回顾性队列研究设计，兼顾了CEA、CYFRA21-1两项TMs，分析其水平的变化对NSCLC患者术后预后的影响。

## 1 资料和方法

### 1.1 临床资料

选取2006年6月-2014年3月首都医科大学附属北京友谊医院胸外科收治的175例NSCLC患者。纳入标准：①均接受肺癌手术切除治疗的患者。②均接受术后辅助4周期化疗（其中Ib期92例患者伴有胸膜受侵、肿瘤直径≥4 cm或者脉管里可见癌栓等高危因素）；均采用双药化疗方案：卡铂/顺铂+紫杉醇/吉西他滨/培美曲塞。③均有pTNM分期（国际肺癌研究协会肺癌分期^[4]^）和术后病理类型[2004年世界卫生组织（World Health Organization, WHO）肺癌组织学类型分型^[8]^]；④均在术前1周内化验CEA和CYFRA21-1，术后2个月内行第1次辅助化疗前复查CEA和CYFRA21-1。排除标准：①经开胸探查活检或者计算机断层扫描（computed tomography, CT）引导下针刺活检等未能切除肿瘤的患者。②术后辅助化疗次数小于4周期的患者或者接受过靶向治疗的患者。③术后分期为Ia期（未化疗）或者临床晚期未能手术治疗的患者。④术前遗漏化验CEA、CYERA21-1或者术后首次化疗前未复查CEA、CYERA21-1的患者。总生存时间（overall survival, OS）指患者手术后第1天到随访为死亡患者的死亡时间或者到末次随访日期（截止日期2014年11月15日）。截止2014年11月15日，154例患者得到随访，随访时间范围为6个月-105个月，平均55.5个月，截止随访日期154例患者中26例死亡，128例存活。此外19例失访，2例拒访，经独立样本t检验，有无失访患者的术前CEA、CYFRA21-1和术后CEA、CYFRA21-1四项指标均无统计学差异（*P* > 0.05）。

### 1.2 材料

154例患者每次化验抽取空腹静脉血3 mL，标本经4, 000 r/min离心分离出血清后，直接检测，CYFRA21-1采用电化学发光法，仪器为罗氏公司提供的Roche电化学分析仪，临界值为3.30 ng/mL；CEA采用化学发光免疫测定法，试剂盒由美国雅培公司生产，临界值为5 ng/mL。

### 1.3 方法

依据术前血清CEA水平分为术前CEA正常组（N）106例（68.83%）和术前CEA升高组（H）48例（31.17%）。依据术前血清CYFRA21-1水平分为术前CYFRA21-1正常组（N）93例（60.39%）和术前CYFRA21-1升高组（H）61例（39.61%）。依据术前术后血清CEA水平分为术前术后CEA均正常组（NN）105例（68.18%）、术前正常术后升高组（NH）1例（0.65%）、术前升高术后正常组（HN）31例（20.13%）、术前术后均升高组（HH）17例（11.04%）。依据术前术后血清CYFRA21-1水平分为术前术后CYFRA21-1均正常组（NN）85例（55.19%）、术前正常术后升高组（NH）8例（5.20%）、术前升高术后正常组（HN）46例（29.87%）、术前术后均升高组（HH）15例（9.74%）。依据术前术后血清CEA联合术前术后CYFRA21-1水平分为术前术后CEA联合术前术后CYFRA21-1均正常组（NNNN）60例（38.96%）、有3项正常1项升高组（NNNH）56例（36.36%）、有2项正常2项升高组（NNHH）32例（20.78%）、术前术后CEA联合术前术后CYFRA21-1均升高组（HHHH）6例（3.90%）（注：正常系指标水平低于临界值，升高系指标水平等于或者高于临界值）。

### 1.4 统计学方法

所有数据采用统计软件SPSS 20.0分析。独立样本*t*检验比较有无失访数据的差异性，*Kaplan-Meier*法对各组进行生存分析，行*Log-rank*检验。Cox比例风险回归模型用来分析预后相关的危险因素，*P* < 0.05认为差异有统计学意义。

## 2 结果

### 2.1 患者基本资料

154例有完整记录的NSCLC患者中，平均年龄61岁（范围：40岁-79岁）。男性96例，女性58例。手术切除的方式为肺叶切除141例，一侧全肺切除4例，楔形切除9例。病理证实为腺癌97例，鳞癌52例，其他类型癌（如腺鳞癌）5例。病理分期Ⅰ期92例，Ⅱ期34例，Ⅲ期28例（表 1）。

**1:** 154例随访患者的临床资料总结 Summary of clinical data of 154 patients with follow-ups

Parameter	*n* (%)
Age (yr)	
≥65	54 (35.06)
< 65	100 (64.94)
Mean (range)	61 (40-79)
Gender	
Male	96 (62.34)
Female	58 (37.66)
Operative procedures	
Lobectomy	141 (91.56)
Over lobectomy	4 (2.60)
Wedge resection of lung	9 (5.84)
Histology	
Adenocarcinoma	97 (62.99)
Squamous-cell carcinoma	52 (33.77)
Other	5 (3.24)
pTNM	
Ⅰb	92 (59.74)
Ⅱa	27 (17.53)
Ⅱb	7 (4.55)
Ⅲa	25 (16.24)
Ⅲb	3 (1.94)
TNM: tumor-node-metastasis.	

### 2.2 术前CEA、CYFRA21-1的水平变化对NSCLC患者术后的生存影响的分析结果

CEA（H）和CEA（N）组患者5年生存率分别为62%和77%，但差异无统计学意义（*P*=0.069）。CYFRA21-1（H）和CYFRA21-1（N）组患者5年生存率分别为55%和84%，差异有统计学意义（*P*=0.001）（图 1）。

**1:**
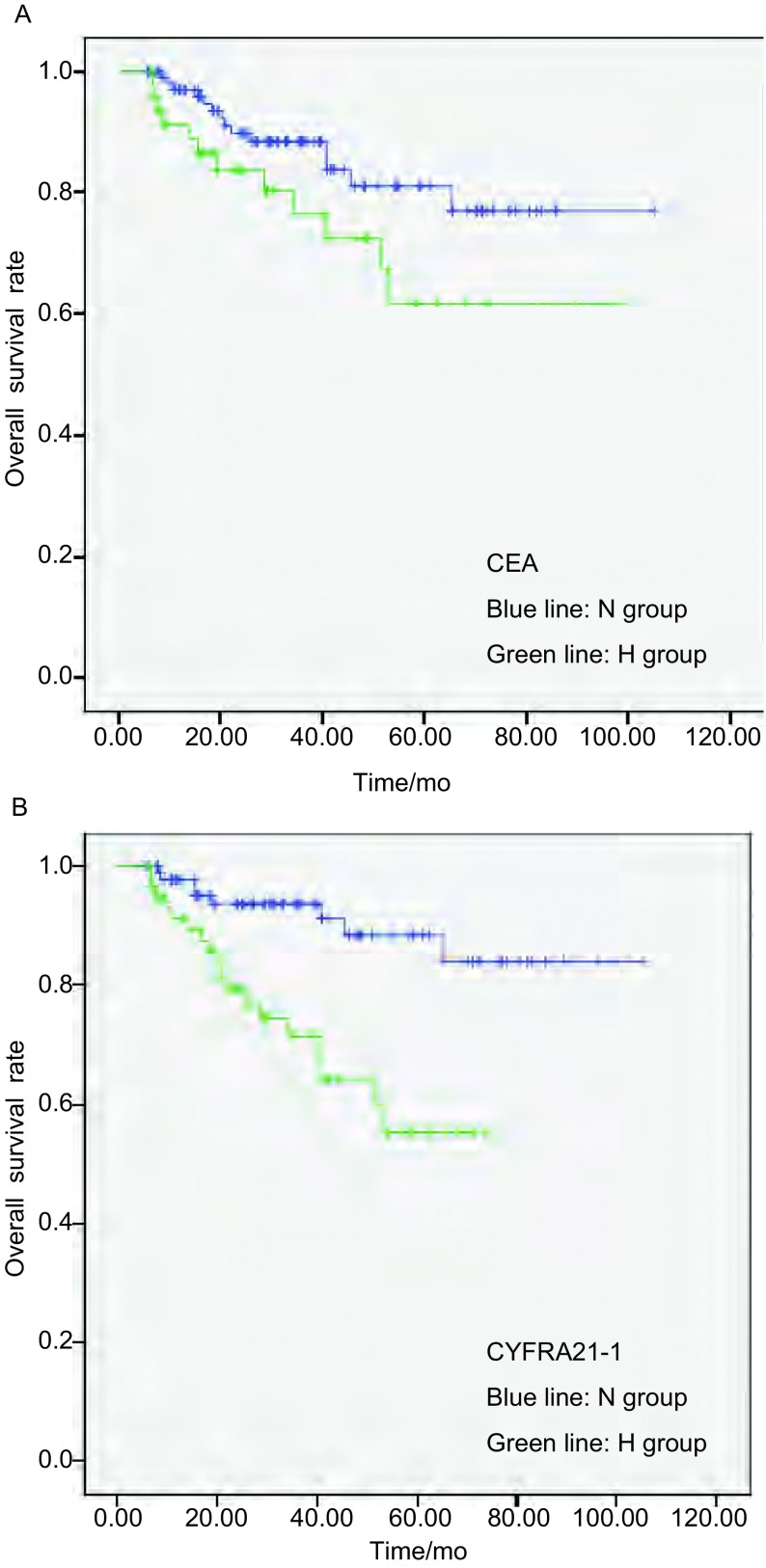
术前CEA、CYFRA21-1的水平变化对NSCLC患者术后的生存影响。A：术前CEA（N）组、术前CEA（H）组患者的生存曲线均呈下降趋势，但是后者下降不明显（*P*=0.069）。B：术前CYFRA21-1（N）组、术前CYFRA21-1（H）组患者的生存曲线均呈下降趋势，后者下降明显，表示其预后不良（*P*=0.001）。 Analysis of survival impact of preoperative CEA and CYFRA 21-1 level variances on patients with NSCLC after surgery. A: Survival curves of the groups of patients with normal preoperative CEA and high preoperative CEA displayed decreasing trends. The decreasing of the latter was not significant (*P*=0.069). B: Survival curves of the groups of patients with normal preoperative CYFRA21-1 and high preoperative CYFRA21-1 displayed decreasing trends. The decreasing of the latter was significant (*P*=0.001) which means poor prognosis. CEA: carcinoembryonic antigen; CYFRA21-1: soluble fragment of cytokeratin-19; NSCLC: non-small cell lung cancer.

### 2.3 术前术后CEA、CYFRA21-1的水平变化对NSCLC患者术后的生存影响的分析结果

CEA（HH）、CEA（HN）、CEA（NN）组患者5年生存率分别为36%、69%、78%，差异有统计学意义（*P*=0.001）。CYFRA21-1（HH）、CYFRA21-1（HN）、CYFRA21-1（NN）组患者5年生存率分别为33%、65%、88%，差异有统计学意义（*P* < 0.001）[注：CYFRA21-1（NH）组8例，统计学未能分析出5年生存率）（图 2）]。

**2:**
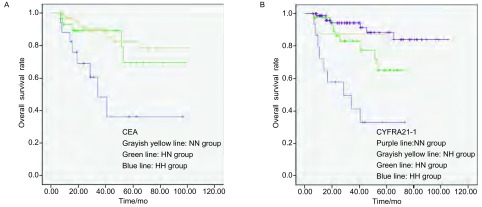
术后CEA、CYFRA21-1的水平变化对NSCLC患者术后的生存影响。A：和其它2组比较，CEA（HH）组患者的生存曲线下降最为明显，表示其预后最差（*P*=0.001）。B：和其他3组比较，CYFRA21-1（HH）组患者的生存曲线下降最为明显，表示其预后最差（*P* < 0.001）。注: CEA（NH）组仅1例，已删除。 Analysis of survival impact of preoperative and postoperative CEA or CYFRA 21-1 level variances on patients with NSCLC after surgery. A: Compared with the other two groups, the survival curve of the group of patients with high preoperative and postoperative CEA levels was significantly steepest (*P*=0.001) while declining, which means that its prognosis was worst; B: Compared with the other three groups, the survival curve of the group of patients with high preoperative and postoperative CYFRA21-1 levels was significantly steepest (*P* < 0.001) while declining, which means that its prognosis was worst. Note: CEA (NH) group was deleted since this group just contained 1 case.

### 2.4 术前术后CEA联合术前术后CYFRA21-1的水平变化对NSCLC患者术后的生存影响的分析结果

术前术后CEA联合术前术后CYFRA21-1（HHHH）组、（NNHH）组、（NNNH）组、（NNNN）组患者5年生存率分别为17%、49%、79%、90%，差异有统计学意义（*P* < 0.001）（注：术前术后CEA联合术前术后CYFRA21-1中有1项正常3项升高即NHHH组3例，统计学效率低，合并于NNHH组）（图 3）。

**3:**
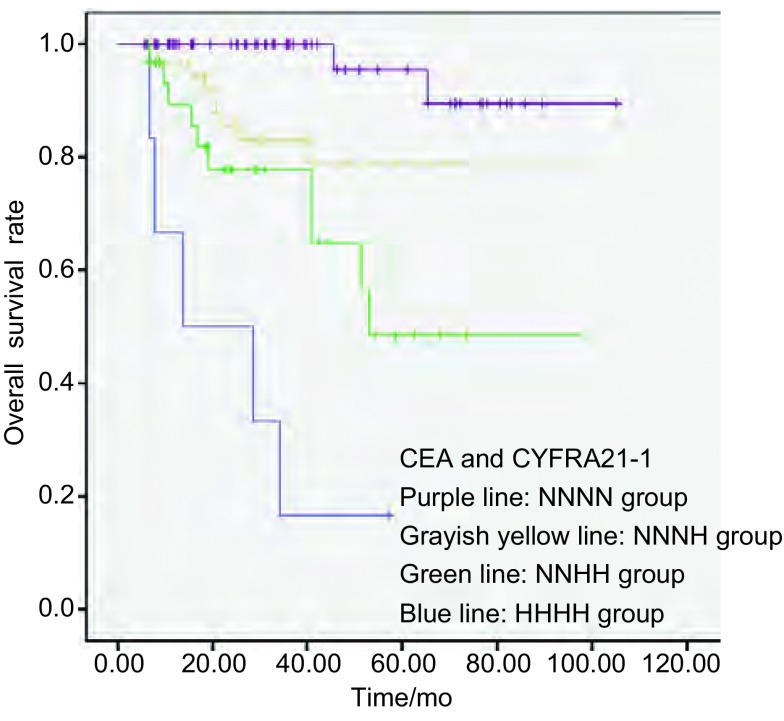
术前术后CEA联合术前术后CYFRA21-1的水平变化对NSCLC患者术后的生存影响和其它3组比较，术前术后CEA联合术前术后CYFRA21-1（HHHH）组患者的生存曲线下降最为明显，表示其预后最差（*P* < 0.001）。 Analysis of survival impact of preoperative and postoperative CEA and CYFRA 21-1 level variances on patients with NSCLC after surgery. Compared with the other three groups, the survival curve of the group of patients with high preoperative and postoperative CEA and CYFRA21-1 levels was significantly steepest (*P* < 0.001) while declining, which means that its prognosis was worst.

### 2.5 影响NSCLC患者术后预后因素的单因素分析

性别（HR=4.699, 95%CI: 1.401-15.556）、pTNM分期（HR=1.739, 95%CI: 1.090-2.774）、CYFRA21-1（HR=1.016, 95%CI: 1.002-1.029）为影响OS的预后危险因素（表 2）。

**2:** NSCLC患者术后预后影响因素的单因素分析结果 The results of univariate analysis of impacting factors of the prognosis for patients with NSCLC after surgery

Parameter	OS	
	HR (95%CI)	*P*
Age	1.044 (0.474-2.302)	0.914
Gender	4.669 (1.401-15.556)	0.012
Operative procedures	1.270 (0.636-2.533)	0.498
Histology	1.033 (0.487-2.191)	0.932
pTNM	1.739 (1.090-2.774)	0.020
CEA	1.004 (1.000-1.009)	0.063
CYFRA21-1	1.016 (1.002-1.029)	0.021
OS: overall survival.		

### 2.6 影响NSCLC患者术后预后因素的多因素分析

依据单因素结果（性别、pTNM分期、CYFRA21-1）和临床考虑有意义的因素（年龄）分别和重点研究的指标，即手术前后CEA各分组、CYFRA21-1各分组和手术前后CEA联合手术前后CYFRA21-1各分组入组多因素分析：男性（HR=0.240, 95%CI: 0.065-0.887）（HR=0.224, 95%CI: 0.066-0.904）、CEA（HH）（HR=4.128, 95%CI: 1.527-11.157）、CYFRA21-1（HH）（HR=6.090, 95%CI: 2.113-17.549）、CEACYFRA21-1（NNHH）（HR=8.335, 95%CI: 1.733-40.089）、CEACYFRA21-1（HHHH）（HR=43.159, 95%CI: 8.020-232.250）为影响OS的独立危险因素。表明男性的死亡风险高于女性，CEA（HH）、CYFRA21-1（HH）、CEACYFRA21-1（NNHH）和CEACYFRA21-1（HHHH）组的患者死亡风险高于各自分组中的患者，其中又以CEACYFRA21-1（HHHH）组的患者死亡风险最高，是CEACYFRA21-1（NNNN）组患者死亡风险的43.159倍（注：CYFRA21-1为控制变量）（表 3）。

**3:** NSCLC患者术后预后影响因素的多因素分析结果 The results of multivariate analysis of impacting factors of the prognostic for patients with NSCLC after surgery

Parameter	OS		Parameter	OS	
	HR (95%CI)	*P*		HR (95%CI)	*P*
Age (≥65 *vs* < 65)	1.500 (0.627-3.590)	0.363	Age (≥65 *vs* < 65)	1.544 (0.640-3.727)	0.334
Gender (Male *vs* Female)	0.240 (0.065-0.887)	0.032	Gender (Male *vs* Female)	0.224 (0.066-0.904)	0.035
pTNM (Ⅱ *vs* Ⅰ)	0.690 (0.245-1.943)	0.483	pTNM (Ⅱ *vs* Ⅰ)	0.716 (0.253-2.032)	0.531
pTNM (Ⅲ *vs* Ⅰ)	1.960 (0.740-5.188)	0.175	pTNM (Ⅲ *vs* Ⅰ)	2.022 (0.768-5.323)	0.154
CEA (HH *vs* NN)	4.128 (1.527-11.157)	0.005	CEACYFRA21-1 (HHHH *vs* NNNN)	43.159 (8.020-232.250)	< 0.001
CEA (HN *vs* NN)	1.403 (0.440-4.476)	0.567	CEACYFRA21-1 (NNHH *vs* NNNN)	8.335 (1.733-40.089)	0.008
CYFRA21-1 (HH *vs* NN)	6.090 (2.113-17.549)	0.001	CEACYFRA21-1 (NNNH *vs* NNNN)	3.605 (0.755-17.223)	0.108
CYFRA21-1 (HN *vs* NN)	2.107 (0.731-6.609)	0.167			
CYFRA21-1 (NH *vs* NN)	2.220 (0.254-19.377)	0.471			

## 3 讨论

如何预测NSCLC患者术后哪些患者预后差，进而予以积极治疗，以提高术后生存率，是我们目前面临的重大难题。至今，许多生物学标志被证实可以判断术后NSCLC患者的预后，如*k-ras*突变、P53、erbB2/Neu、Bcl-2等^[9-11]^，但这些生物学标志的结果需要通过手术获取标本而实现，付出的成本高、代价大。也有研究^[12, 13]^指出VPI、BVI、pT分期、肿瘤异质性可以预测NSCLC患者术后的预后情况。和上述指标比较，TMs是预测NSCLC患者术后预后的最佳指标，这是由于他们检测的操作方法简便、并且具有可重复性、准确性、成本低等优势^[2, 3]^。

CEA，即癌胚抗原，最初发现于结肠癌，之后发现高表达于肺癌、食管癌等癌^[14]^。有报道^[3]^认为高龄、男性、VPI、pT2期等是引发术前CEA水平高的原因。相反，Wang等^[2, 7]^认为术前CEA水平高仅和肺癌的组织学类型有关。有报道^[1]^认为术后CEA水平升高受肺外疾病、手术不彻底影响。无论术前术后CEA水平升高的原因是什么，比较确切的是，CEA水平的升高传递出一个坏信息，即患者预后差。本研究图 1显示术前CEA（H）组患者的预后差，但差异无统计学意义。与本研究这一结论不同的是，Okada等^[7]^证实了术前CEA（H）是NSCLC患者术后预后的危险因素。进一步分组后分析，和CEA（NN）等组比较，CEA（HH）组患者的预后最差，并且是判断NSCLC患者术后预后的独立危险因素。这一结论和Okada等^[7]^各自研究的结论相一致。

此外，血清CEA水平的变化对预测晚期肺癌患者的疗效和预后都具有一定的价值。Arrieta等^[15]^的研究表明，对给予仅仅1种化疗方案，同时满足CEA水平大于10 ng/mL的晚期NSCLC患者，血清CEA的水平变化（升高或降低）可以预测患者的治疗反应。又如使用埃罗替尼治疗晚期肺癌之前，CEA、CYFRA21-1中1项或者2项水平升高预示着患者预后差^[16]^。CEA水平升高也存在益处，Jung等^[17]^研究指出使用EGFR-TKIs治疗的晚期NSCLC患者，治疗前CEA水平升高会带来良好的治疗效果，这可能由于高水平CEA和活化的EGFR突变之间存在某种联系^[18]^。

CYFRA21-1是细胞角蛋白的可溶性片段，广泛分布在层状或鳞状上皮中^[19]^。术前CYFRA21-1水平升高与pT、N分期密切相关，T分期高及肿瘤越大时，CYFRA21-1水平越高^[19]^，相同，N分期越高，患者CYFRA21-1阳性率也越高^[20]^。这表明早期肺癌患者CYFRA21-1阳性率低，因此他对早期肺癌的筛查作用微小^[19, 21]^。本研究中，154例患者术前CYFRA21-1阳性率为39.61%，高于Suzuki等^[22]^研究中的5.9%和Muley等^[5]^的21.2%，考虑这一差异和各研究中选取的样本不一有关，本研究的样本包含了术后病理为Ⅱ期、Ⅲ期的患者，故CYFRA21-1阳性率偏高。和对早期肺癌筛查的作用微小相反，CYFRA21-1水平变化对预测NSCLC患者预后的作用较大。Suzuki等^[22]^研究显示术前CYFRA21-1水平升高的患者早期死亡风险率高，Myley等^[5]^证实了术前CYFRA21-1水平升高是判断NSCLC患者术后预后的独立危险因素；本研究也证实了这两点。此外，本研究还特别指出：和CYFRA21-1（NN）等组对比，CYFRA21-1（HH）组患者的预后最差，差异有统计学意义，并且是判断NSCLC患者术后预后的独立危险因素。依据术前术后CEA的水平变化情况联合术前术后CYFRA21-1的水平变化情况进行分组进一步分析得出CEACYFRA21-1（HHHH）组和（NNHH）组是判断NSCLC患者术后预后的独立危险因素，所在分组中前者的预后最差，其死亡风险是CEACYFRA21-1（NNNN）组患者死亡风险的43.159倍。

一直以来，pTNM分期是肺癌患者术后判断预后和治疗的主要参考依据^[21]^，本研究的多因素Cox风险比例分析中，pTNM分期为Ⅱ期（22.08%）、Ⅲ期（18.18%）的患者，分别以Ⅰ期（59.74%）患者对照分析，结果表明Ⅱ期、Ⅲ期患者的死亡风险相对于Ⅰ期患者的死亡风险无统计学意义，非判断预后的独立危险因素（P值均大于0.05）。相关研究^[2, 16, 22]^也未得出相应的pTNM分期为判断预后的独立风险因素。这和临床中pTNM分期对预后的显著作用相悖。我们考虑本研究出现这一结果和各分期组中样本比例差距较大有关。此外，我们尝试将Ⅱ期、Ⅲ期患者合并为偏晚期组，与Ⅰ期患者相比，差异有统计学意义（*P*=0.015 < 0.001）。考虑到pTNM分期并不是本文讨论之重点，我们仍按照Ⅱ期、Ⅲ期规范分组分析后做记录。与血清CEA、CYFRA21-1相比较，pTNM分期作为术后治疗的依据存在一定局限性^[7]^，因为CEA、CYFRA21-1更能具体化和准确化NSCLC患者术后的预后情况。比如肺癌术后Ib期患者不推荐化疗，本研究术后Ib期患者共92例（59.74%），其中术前术后CEA或CYFRA21-1均升高的患者各占6例（13.04%），术前术后CEA和CYFRA21-1均升高的患者占2例（2.2%）。由这部分患者的预后结果看，予以积极的治疗和监测，改善生存率是必要的。虽然美国国立综合癌症网络（National Comprehensive Cancer Network, NCCN）不推荐对癌症患者诊疗时常规检查TMs^[2]^，至今一些欧美国家对其重视度尚低^[7]^；但是越来越多研究^[1-3, 5-7, 14-22]^报道了TMs可以预测NSCLC患者的治疗效果及预后。他们的水平变化有助于pTNM分期T分期内部进一步分层，也可能成为术后Ib期NSCLC患者实施辅助化疗的较可靠指征。

综上所述，血清CEA或CYFRA21-1在手术前及术后均高于正常，尤其是两者联合在手术前及术后均升高的NSCLC患者预后显著不良。手术前后血清CEA、CYFRA21-1的检测有助于NSCLC患者术后预后的判断。本研究也存在一定局限：一项回顾性研究；样本量偏少，按照病理类型做亚组等分析时统计学效率低。
